# The Decay of Moral Force

**Published:** 1914-08-01

**Authors:** 


### The Hospital

The Workers' Newspaper of Administrative Medicine and Institutional Life, Administration, National Insurance and Health.

No. 1467, Vol. LVI. SATURDAY, AUGUST 1, 1914. PRICE ONE PENNY.

### THE DECAY OF MORAL FORCE.

There has never been a time like the present in the history of the British people. The country has enjoyed great prosperity of late. Work and money are abundant, but the passion for motion and the unending craving for amusement and relaxation is becoming so excessive as to threaten the nervous energy of individuals to the danger of the race. The fashion is to declaim that there is no time for anything. The truth being that the excessive cultivation of a desire for hurry and rush and motion makes any natural and wholesome enjoyment well-nigh impossible. This state of affairs is undermining the physical powers of both men and women of all classes.

The changed conditions of life here referred to harmfully affect the working members of the race, whatever their occupations may be. The professional man cannot give an opinion of any real value unless he devotes time and knowledge, as the result of experience and hard work, to each case submitted to him. This is true not only of members of the medical profession, but of lawyers and experts in every branch of work throughout the world. Rule-of-thumb methods produce results which are not valuable in a business sense, and there is a growing feeling amongst thoughtful persons that it is more and more difficult to find consultants who do produce opinions which are of the highest value.

In politics the incapacity displayed in the handling of affairs in this country for years past has amazed the world and threatens the very existence of the United Kingdom as one of the Great Powers. The escapades of modern women, their mode of dress, their love to exhibit themselves everywhere in what are known as risky costumes, recalls a state of affairs which in the early days of Napoleon's rule led him to refuse to admit to the Court any woman who failed to display, in regard to both clothes and conduct, the highest self-respect for herself and her sex.

From the hygienic and physical points of view the conduct of the individual, his or her mode of life, the evidence each affords of a standard of personal conduct, or the reverse, have vital influences upon health and the general virility of the race. An increasing number of journalists are not free from exhibiting the prevailing symptoms of physical degeneration which characterise those specimens of our nation who to-day are crazy after notoriety. We remember a man and his wife, of ability and good position, who thirty years ago grew sad indeed if one or both their names did not appear in the *Times* on most days of the week. This craving for mere notoriety is carried to such excess at the present time that it indicates that sober judgment and high personal conduct are failing to carry due weight with masses of the people. These modern crazes have reacted upon certain types of papers which demonstrate such an astounding disregard for facts and truth that the sound influence of the press on public opinion is rapidly diminishing.

All these causes have produced evil effects upon the former high standards of personal conduct and individual responsibility which once made England great. Questions of truth, of honesty, of the just claims of other people, of honour, and of self-respect, not to mention probity, justice, self-restraint, and the higher ideals of life and conduct, are not only disregarded, but ignored by an increasing number of the silliest and most useless members of all classes in the ceaseless whirl and rivalries of modern existence. The same weakening tendencies are exhibited in regard to the enforcement of discipline upon children, the infliction of punishment upon the criminal, and the compulsion of order upon the unruly. The failure to display sober judgment and to inculcate sound principles is becoming a danger to the nation. Trade Unionism, for example, is not content merely to exercise its authority by exalting incompetence at the expense of the ablest and most conscientious members of the working classes. At a meeting of the Federated Trades Councils of Yorkshire, at Shipley, on July 18, the seconder of a resolution ended by calling upon all Trade Unionists "to use all their influence to prevent their offspring from joining the "police and military forces"; and advised "any

" starving workers that before they should go into " the Army to do the dirty work of the capitalistic " classes, they should steal."

We are glad to note that the majority of those present declined to support the proposal that Trade Unionists should become a gang of thieves. They further eliminated the call to their offspring to shed all patriotism. Still, the fact that it is possible for anyone in this country to stand up amongst his fellows and propose and defend the habitual practice of theft, and a concentrated effort to compel the youth of the country to shed their manhood and learn to become hopeless degenerates, is startling evidence of the depths to which the steady destruction of moral force in the individual is being pursued. No healthy, strong, purposeful, self-respecting, wholesome man or woman can find happiness or success, or realise the joys of life and the splendid helpfulness which comes from the habitual following of the path of duty, with the exercise of the higher qualities of humanity, who does not continuously set his or her face against the tide of modern degeneration to which we have called attention. Such degeneration is a symptom of threatening decadence, and unless the men and the women of to-day, who are capable of leadership, being sober-minded and responsible people, will wake up, stand shoulder to shoulder, and suppress the causes which are sapping England's strength, the end of England's glory and influence must come and quickly prove beyond recall.

